# Investigation of SNPs in the porcine desmoglein 1 gene

**DOI:** 10.1186/1746-6148-3-4

**Published:** 2007-03-31

**Authors:** Lise Daugaard, Lars Ole Andresen, Merete Fredholm

**Affiliations:** 1 The National Veterinary Institute, Technical University of Denmark, Bülowsvej 27, 1790 Copenhagen V, Denmark; 2 Faculty of Lifesciences, University of Copenhagen, Bülowsvej 17, 1870 Frederiksberg C, Denmark

## Abstract

**Background:**

Desmoglein 1 (DSG1) is the target protein in the skin disease exudative epidermitis in pigs caused by virulent strains of *Staphylococcus hyicus*. The exfoliative toxins produced by *S. hyicus *digest the porcine desmoglein 1 (PIG)DSG1 by a very specific reaction. This study investigated the location of single nucleotide polymorphisms (SNPs) in the porcine desmoglein 1 gene (PIG)*DSG1 *in correlation to the cleavage site as well as if the genotype of the SNPs is correlated to susceptibility or resistance to the disease.

**Results:**

DNA from 32 affected and 32 unaffected piglets with exudative epidermitis were diagnosed clinically as affected or unaffected. Two regions of the desmoglein 1 gene were sequenced and genotypes of the SNPs were established. Seven SNPs (823T>C, 828A>G, 829A>G, 830A>T, 831A>T, 838A>C and 1139C>T) were found in the analysed sequences and the allele frequencies were determined for the SNPs resulting in amino acid change. Four of the seven polymorphisms were situated in the motif known to be important for toxin cleavage. The distribution of the genotypes between affected and unaffected animals was analysed.

**Conclusion:**

The study indicated a possible correlation between the genotypes of two out of seven SNPs found in the porcine desmoglein 1 gene and the susceptibility to exudative epidermitis.

## Background

Desmoglein 1 is one of the adhesive proteins in the desmosomal complex that forms one of the intercellular junctions found in epithelial tissues [1]. Desmogleins are calcium-dependent transmembrane glycoproteins and members of the cadherin superfamily [2]. Desmoglein 1 is the target protein in the skin disease exudative epidermitis (EE), which is a disease in pigs caused by virulent strains of *Staphylococcus hyicus*. Exfoliative toxins from *S. hyicus *(Exhs) digest porcine desmoglein 1 in the extracellular part, which is responsible for the cell-cell adherence. The digestion of desmoglein 1 causes exfoliation of the skin, which is a characteristic symptom of the disease [3]. Different types of toxins from *S. hyicus *have been identified. ExhA, ExhB, ExhC and ExhD have been identified in Denmark and can be distinguished by PCR [4]. Another related exfoliative toxin SHETB has been identified in Japan [4,5].

**Figure 1:**
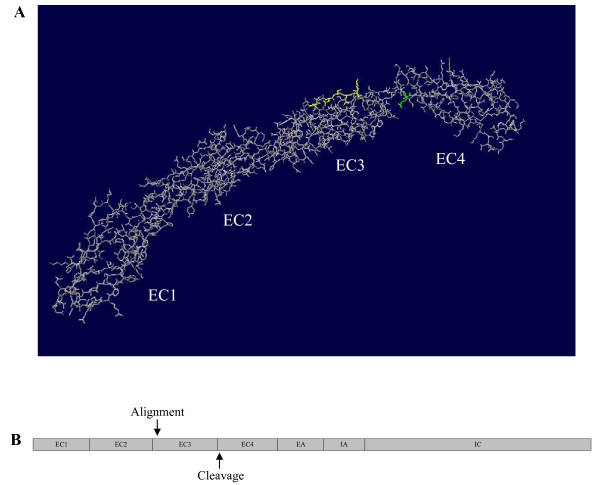
**Structure of the porcine desmoglein 1 gene and gene product**. **A**, The four extracellular domains (EC1-4) of the porcine desmoglein 1. (PIG)DSG1 is modelled on top of the crystal structure of C-cadherin. The 3D structure indicates that the cleavage site is situated between EC3 and EC4 [7] (green). The amino acids of the important loop in human sequence is also in the porcine structure placed on a loop on the opposite side of EC3 (yellow) [8]. **B**, The arrangement of the porcine desmoglein 1 protein. The cleavage site and the putative alignment motif are marked with arrows in the extracellular domains. EA: Extracellular anchor domain, IA: Intracellular anchor domain, IC: Intracellular domain. [7]

Human desmoglein 1 ((HUMAN)DSG1) is known to be the target for the exfoliative toxins (ETs) from *S. aureus *involved in the disease staphylococcal scalded skin syndrome (SSSS) [6]. In figure 1 the schematic form of the domain structure and a modelling of the 3D structure for the porcine desmoglein 1 protein is shown. Since the porcine desmoglein 1 (GenBank: AAV84914) is homologous to the human desmoglein 1 (GenBank: AAC83817) (81.9% amino acid sequence identity [7]), it is assumed that the cleavage site for the exfoliative toxins and other properties of the porcine desmoglein 1 are similar to those of the human desmoglein 1. Both the human and the porcine desmoglein 1 are believed to have a structure similar to classical cadherins with four repeating extracellular domains stabilised by Ca^2+ ^ions. The cleavage site is situated between the third and the fourth extracellular domain close to a calcium-binding site [6] as indicated in figure 1.

The reaction between ETs and human desmoglein 1 is highly specific and dependent on not only the amino acid sequence, but also the calcium-stabilised three-dimensional structure [8]. A motif of five amino acids in the human desmoglein 1 sequence, situated approximately 110 amino acids upstream of the cleavage site has been shown to be necessary for cleavage by the exfoliative toxin (ETA) from *S. aureus*. The motif is proposed to be involved in the alignment of desmoglein 1 to the toxin during cleavage. In the same study it is suggested that binding of desmoglein to the toxin and cleavage are independent abilities [8]. Structure prediction shows that these amino acids are placed on a loop on the surface of extracellular domain 3 [8] (figure 1, panel A). A shared mechanism has been proposed for the toxin cleavage of desmoglein 1 in the human disease SSSS and the porcine disease EE [3].

Between individual piglets in a litter affected by EE large differences in the severity of the disease can occur, as some animals will develop severe exfoliation of the skin on most of the body, while other individuals in the same litter only show local or no symptoms. Since desmoglein 1 is the target protein for the exfoliative toxins, it is possible that SNPs in the gene encoding the porcine desmoglein 1 could be important for the susceptibility/resistance to EE in piglets.

This study investigated if polymorphisms in the porcine desmoglein 1 are found at the cleavage site and in the sequence motif upstream to the cleavage site homologous to the motif in the human sequence known to be necessary for cleavage by exfoliative toxins from *S. aureus*. Since the extracellular domains of the porcine and the human desmoglein 1 proteins share significant homology [7] it is likely that the amino acids in the upstream domain of the porcine protein also are important for binding of the Exh toxins from *S. hyicus*. Therefore, the genotypes of affected and unaffected piglets from herds with EE were analysed, in order to establish a possible correlation between a certain genotype and susceptibility or resistance to the disease.

## Results

To investigate polymorphisms in the porcine desmoglein 1 gene and analyse if the genotype is correlated to susceptibility towards EE, blood samples from affected and unaffected animals were collected and their genotypes were established by sequencing.

### Diagnosis

The blood samples from affected and unaffected piglets were collected in two Danish pig herds. A total of 32 affected piglets and 32 unaffected piglets (table 1) were clinically diagnosed. Difference in extent of symptoms was observed in the group of affected piglets, but all animals with EE symptoms were designated as affected. Piglets designated as unaffected had no symptoms. Blood samples and skin swabs were collected from all the diagnosed piglets and from 11 sows. Multiplex PCR for toxin ExhA, B, C and D on 5 *S. hyicus*-like colonies from each animal verified that toxin positive bacteria were present on all animals except for one litter, which confirmed that EE was the disease causing the symptoms of the affected animals. In herd 1, three different types of exfoliative toxins (ExhA, ExhB and ExhD) were found and in herd 2, two different toxin types (ExhA and ExhC) were found (table 1). In all cases only clones positive for one toxin were found on the individual animal, except for one piglet having both ExhA and ExhC positive clones.

### Polymorphisms

**Figure 2:**
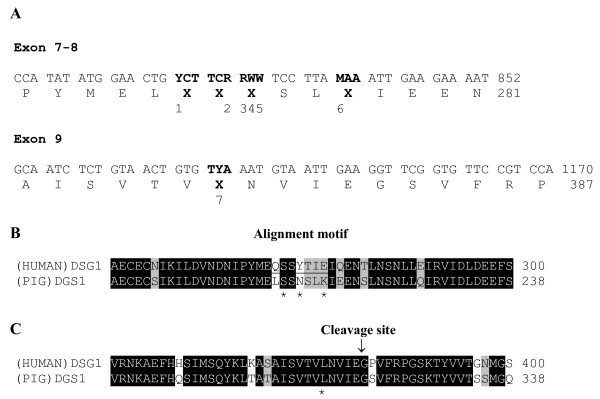
**Amino acid changing polymorphisms in two areas of the porcine desmoglein 1 gene**. **A**, Degenerate sequences of the porcine desmoglein 1 in the investigated regions showing part of the fragments amplified in the genotyping procedure. SNPs are marked with X. R: A or C, W: A or T, M: A or C, Y: C or T. **B**, Amino acid sequence alignment of human (HUMAN)DSG1 GenBank: AAC83817 and porcine (PIG)DSG1 GenBank: DQ823081 desmoglein 1 in the region around 110 amino acids upstream of the cleavage site. Underlined in the human sequence are the amino acids shown to be necessary for cleavage by exfoliative toxin ETA from *S. aureus*. Marked with asterisks * in the porcine sequence are the residues changed by single nucleotide polymorphisms found in this study. **C**, Alignment of (HUMAN)DSG1 and (PIG)DSG1 around the cleavage site (marked with arrow). Marked with asterisk * in the porcine sequence is the amino acid changed by single nucleotide polymorphism.

Two fragments of the porcine desmoglein 1 gene were amplified using PCR and sequenced from a total of 75 animals (sows and piglets). Primers used for amplification are listed in table 4. The first fragment of 511 bp covers a part of exon 7, intron 7 and a part of exon 8. The amino acid sequence translated from this DNA-sequence upstream to the cleavage site is assumed to be necessary for cleavage by Exh toxins [8]. The other fragment of 165 bp covers a part of exon 9. The amino acid sequence translated from exon 9 covers the area around the cleavage site. Figure 2, panel A shows part of the fragments amplified by the PCR method.

The nucleotide sequences generated from all sampled animals were inspected and aligned to establish amino acid changes in the upstream domain and around the cleavage site. The sequences were also compared to the cloned sequences of porcine desmoglein 1 (Acc. Number in GenBank: AAV84914, DQ823081 and DQ823082, data not shown).

Six SNPs were found in the upstream region and one in the region close to the cleavage site (number 1–7 in figure 2, panel A). The SNPs and their corresponding codons are listed in table 2. SNP number 1 is a missense mutation 823T>C changing the resulting amino acid from serine to proline. Among both affected and unaffected animals genotyped only homozygous P/P and the heterozygous P/S individuals were identified. SNP number 2 is a silent mutation 828A>G with no change of amino acid residue. Number 3, 4 and 5 are also missense mutations 829A>G, 830A>T, 831T>A changing the codon from AAT (asparagine) to GTA (valine). Only these two combinations of the codon were found and only the version containing the codon for valine was present among the animals genotyped from the herds. The version containing the codon for asparagine was seen in the cloned sequences from Denmark (accession number in GenBank: DQ823081 and DQ823082) and Japan (accession number in Genbank: AAV84914). SNPs number 6 and 7 are also missense mutations 838A>C and 1139C>T resulting in changes between lysine and glutamine and leucine and serine respectively. For SNPs number 6 and 7 both homozygous and heterozygous animals were found among the genotyped animals.

To evaluate the probability of the polymorphisms influencing the properties of the protein structure, the two alternative amino acids of the SNPs were compared according to Grantham's physiochemical distances [9]. On the Grantham scale the most similar pair (L to I) has index 5 and the most dissimilar pair (C to F) has index 205. The amino acid pairs of this study have the indices 74, 0, 133, 53 and 145 (table 2).

Allele frequencies were calculated for the alleles of SNPs resulting in amino acid change (number 1, 6 and 7). The frequencies are based on genotypes of animals from both herds including the sows. Thus, they might not reflect the true allele frequencies in the population, since some of the animals are related. For SNP number 1 P has a frequency of 0.86 and S a frequency of 0.14. For SNPs number 3, 4 and 5 frequencies are not calculated because only one genotype (GTA) was found in the material from the two herds. For SNP number 6 the frequencies for Q and K are 0.30 and 0.70, respectively, and for SNP number 7 the frequencies for L and S are 0.43 and 0.57, respectively.

To investigate if there is a correlation between the genotype of the four SNPs resulting in amino acid change in the different piglets and their susceptibility towards EE, the number of affected and unaffected piglets with each genotype was counted and the percentage was calculated. The results are shown in table 3. For SNP 1 an almost equal number of affected and unaffected animals were found for the genotypes P/P and P/S. For SNP 6 there are more affected (28%) than unaffected (9%) animals having genotype Q/Q and more unaffected (69%) than affected (47%) having genotype K/K. For the heterozygous genotype numbers are similar (25 and 22%). For SNP 7 there are more affected (25%) than unaffected (9%) animals having genotype L/L and more unaffected (44%) than affected (25%) having genotype S/S. Also for this locus the heterozygous genotype numbers are similar (50% and 47%).

The number of affected and unaffected animals for each locus was tested statistically (χ^2^-test) to establish whether the differences in numbers for each genotype between affected group the unaffected group were statistically significant. In the first test (χ^2^-test 1, table 3) only the homozygous animals are included. This test is performed on SNP 6 and 7, as no homozygous animals were present for the S allele at SNP 1. χ^2^-test 1 shows that the differences between the homozygous numbers for SNP 6 and 7 are significant at the 95% level. The second test (χ^2^-test 2, table 2) was performed including homozygous and heterozygous numbers. This test was performed on locus 1, 6 and 7 and showed that the differences obtained were not statistically significant.

## Discussion

### SNPs are found in both sequence motifs

Previous data indicate that five amino acids in the region upstream of the cleavage site in the human desmoglein 1 are required for cleavage by the ETA toxin from *S. aureus*. These amino acids are placed on a loop, which is suggested to take part in a specific interaction between the exfoliative toxin and desmoglein 1. The interaction allows for activation of the toxin and cleavage of desmoglein 1 [8] possibly by rearrangement of the oxianion hole near the catalytic site of the exfoliative toxin [10]. The authors responsible for the investigation of the human desmoglein 1 propose that the loop with the five amino acids may be important for proper alignment of the toxin [8]. Therefore we hypothesized if the homogeneously placed amino acids in the porcine desmoglein 1 protein could have importance for the cleavage and binding by the toxins from *S. hyicus*. Modelling of the porcine amino acid sequence on the homologous C-cadherin structure show, that the motif is located on a loop on the surface of extracellular domain 3 (figure 1 panel A), similar to the human desmoglein 1.

In the present study, seven polymorphisms were identified in the two regions of the gene that were investigated. Five of the SNPs (1, 3, 4, 5 and 6) are located among nucleotides coding for the five amino acids, which may be important for toxin cleavage and binding (figure 2 panel B). It is possible that changing an amino acid in this motif could influence the ability of the Exh toxins to bind to the porcine desmoglein 1. With respect to the SNPs 3, 4 and 5 only the codon coding for valine (V) was seen in the sequences of the investigated pigs. The codon coding for asparagine (N) is seen only in the cloned sequences from Denmark GenBank: DQ823081 and DQ823082 and Japan Genbank: AAV84914. This could indicate that the N allele is much less frequent than the V allele. This could be investigated by genotyping a larger group of unrelated animals. For the SNPs 6 and 7, both homozygous and heterozygous animals were present in the two herds. For SNP number 1 (P to S), the homozygous version S/S was not found among the animals from the two herds. This is in accordance with the allele frequencies detected (P: 0.86 and S: 0.14), giving an expected frequency of 1–2 animals homozygous for S in a population of 75 animals.

### Distribution of genotypes between affected and unaffected

There seems to be more homozygous Q/Q and L/L in the affected group and more homozygous K/K and S/S in the unaffected group (table 3). The differences in homozygous numbers were tested excluding the heterozygous numbers, since a putative correlation to the genotype would be easier to distinguish, as the homozygous animals only express one amino acid for each locus. Since it is not known whether the possible susceptibility is a dominant or recessive property both calculations were made. If the property is dominant it will be an advantage to include the heterozygous animals, whereas if the property is recessive, only the homozygous animals will have the property and the heterozygous animals will add statistical noise to the result.

The differences are significant only when the homozygous animals are analysed. When the heterozygous animals are left out of the analysis the total number of animals gets smaller and thereby the evidence becomes weaker. Thus, there is only a weak indication of a correlation between the genotype in SNP 6 and 7 and the susceptibility to EE. However, since the significance disappears when the heterozygous animals are included a simple relationship between a specific mutation and the susceptibility to EE is not present. To be able to investigate this possible correlation further, a larger group of affected and unaffected animals is needed to increase the number of homozygous samples. A possible uncertainty of the result could be that it is not known if all susceptible animals have been under a sufficiently large contagious pressure to get infected. The physicochemical distance between L and S (145) and between N and V (133) (table 2) indicates that the SNPs 3, 4, 5 and 7 could influence the properties of the porcine desmoglein 1 protein.

Other parts of the desmoglein 1 protein might also have influence on the binding and/or activation of the exfoliative toxins. Thus, although this study investigated SNPs in the parts of the desmoglein 1 gene that have been described to be of importance for cleavage by exfoliative toxins, there might be additional sites of importance which have not been included here.

Several types of toxin positive *S. hyicus *clones were involved in each herd (table 1). It is not known if desmoglein 1 with different alleles of the investigated SNPs has different affinity towards the various toxins types. To investigate this further, samples from herds infected only with clones producing one toxin type would be necessary.

## Conclusion

From the results obtained, it can be concluded that SNPs resulting in amino acid changes have been found in seven loci in the investigated regions of the desmoglein 1 gene. Two of the SNPs are situated in a motif homologous to one in the human desmoglein 1 known to be important for exfoliative toxin binding and cleavage. One SNP is close to the cleavage site. For the SNP at this locus, the change from S to L could have an influence on the properties of the desmoglein 1 protein. The study might indicate a correlation between the genotype of SNP 6 and 7 and the susceptibility to exudative epidermitis but further investigations are needed in order to confirm this.

## Methods

### Diagnosis and sample preparation

The clinical diagnosis of the piglets was based on the appearance of their skin lesions and the piglets were categorised as affected or unaffected. Affected piglets all had typical lesions of EE behind the ears and around the eyes and the snout. In more severe cases the lesions had spread to other parts of the body. Piglets classified as unaffected had no skin symptoms. Samples were taken from both affected and unaffected piglets and from some of sows in the herd. Seventy-five blood samples were collected in two herds (1 and 2) infected with EE. Among the 46 animals from herd number 1, 19 piglets were affected, 21 were unaffected and 6 were sows. In herd number 2, 29 samples were taken: 13 affected piglets, 11 unaffected and 5 sows. Some of the piglets are offspring from these sows; however only few of the piglets are full sibs because mixed sperm has been used for insemination. From each animal a blood sample was taken from the jugular vein and a skin swab was obtained from the skin behind the ear. The diagnosis was verified by identification of toxin positive *S. hyicus *bacteria from the skin swabs. Skin swabs from the animals were suspended in 0.9% NaCl and plated on selective and indicative plates [11]. Five *S. hyicus*-like colonies from each animal were chosen for toxin determination by multiplex PCR as described in [4]. From the EDTA stabilised blood samples genomic DNA was extracted using a salting out procedure [12]. The genomic DNA was resuspended in sterile water and diluted to a concentration of 25 ng/ul.

### PCR and sequencing

PCR and DNA sequencing with two sets of primers identified the genotypes of the SNPs. One primer set was designed to amplify a 511 bp fragment covering part of exon 7, intron 7 and part of exon 8. The other primer set was designed to amplify a 165 bp fragment in exon 9. Primer sequences are listed in table 4. Primers were designed using the porcine desmoglein 1 sequence GenBank: DQ823081.

All genomic DNA samples were amplified by PCR for 30 cycles of 94°C for 30 sec., 50°C for 30 sec., 68°C for 30 sec. with both primer sets by standard PCR conditions (60 mM Tris-SO_4_(pH 9.1),18 mM (NH_4_)_2_SO_4_, 1.7 mM MgSO_4_, 20 pmol of each primer, 200 uM each of the four deoxynucleotides and 0.5 μl Elongase Enzyme Mix (Invitrogen, Carlsbad, USA) in 50 μl reactions). 30 ng of genomic DNA was in each reaction. The PCR products were purified using QIAquick spin columns (Qiagen, Hilden, Germany) and sequenced on both strands (by MWG, Ebersberg, Germany). Sequence data were inspected with BioEdit Sequence Alignment Editor v. 7.0.5.3 [13].

### Modelling

Modelling of the extracellular domains of porcine desmoglein 1 3D structure was made using the prediction server CPHmodels [14]. The amino acid sequence of porcine desmoglein 1 GenBank: DQ823082 was used and the prediction server proposes the crystal structure of C-cadherin (PDB ID: IL3W) [15] as a template (score: 189, E: 5e-48). The C-cadherin crystal structure is then used to predict the 3D structure of porcine desmoglein 1 by profile-profile alignment.

### Statistics

A χ^2 ^test was applied to the data set. Data for each SNP was tested separately using the affected and unaffected groups as factors against the possible genotypes. χ^2 ^test 1 was performed on SNP 6 and 7 using only the two homozygous groups (2 × 2 tables). This test could not be used for SNP 1 because the homozygous S/S group was empty. Yates correction was applied to the 2 × 2 tables. χ^2 ^test 2 was performed on SNPs 1, 6 and 7 using all three genotypes (2 × 3 tables).

### Accession numbers

The used sequences have the following accession numbers: Human desmoglein 1 GenBank: AAC83817, porcine desmoglein 1 cloned in Japan GenBank: AAV84914 and Denmark GenBank: DQ823081 and DQ823082.

## List of abbreviations used

SNP: single nucleotide polymorphism

Exh: exfoliative toxin from *Staphylococcus hyicus*

ET: exfoliative toxin from *Staphylococcus aureus*

(HUMAN)DSG1: human desmoglein 1

(PIG)DSG1: porcine desmoglein 1

SSSS: staphylococcal scalded skin syndrome

EE: Exudative epidermitis

PCR: polymerase chain reaction

EC: extracellular domain

IA: Intracellular anchor domain

IC: Intracellular domain

## Authors' contributions

LD planed and conducted the sample collection, clinical evaluation, and all the laboratory work and data analysis of this study and drafted the manuscript. LOA assisted in planning and design of the study, partook in sample collection and clinical evaluation assisted in the data evaluation and helped to draft the manuscript. MF assisted in planning and design of the study, in the data evaluation and helped to draft the manuscript. All authors read and approved the final manuscript.

## Figures and Tables

**Table 1:** Animals tested and sampled in the study

**Herd**	Sows*	**Affected piglets**	**Unaffected piglets**	**Identified toxins**
1	6	19	21	Exh A, B, D
2	5	13	11	Exh A, C
				
Total	11	32	32	

* Some of the sampled piglets are offspring from these sows. Not all are full sibs because mixed sperm was used for insermination.

**Table 2:** SNPs found in the investigated fragments of porcine desmoglein 1

**SNP number**	Nucleotide number *^1^	**Base 1**	**Base 2**	**Codon 1**	**Amino acid 1**	**Codon 2**	**Amino acid 2**	Similarity *^2^
1	823	T	C	TCT	S (Ser)	CCT	P (Pro)	74
2	828	A	G	TCA	S (Ser)	TCG	S (Ser)	0
3	829	A	G					
4	830	A	T	AAT	N (Asn)	GTA	V (Val)	133
5	831	A	T					
6	838	A	C	AAA	K (Lys)	CAA	Q (Gln)	53
7	1139	C	T	TTA	L (Leu)	TCA	S (Ser)	145

*^1 ^Numbering based on full cDNA sequence GenBank: AY677210

*^2 ^Degree of similarity between two amino acids based on physiochemical differences given according to Grantham [9]

**Table 3:** Statistics on numbers of affected and unaffected animals with each genotype

**SNP**	**Genotype**	**Affected number**	**Affected %**	**Unaffected number**	**Unaffected %**	**χ^**2 **^test 1 *p*^**1#)**^**	**χ^**2 **^test 2 *p*^**2#)**^**
1	P/P	25	78	24	75	not det.	not sign.
	S/S	0	0	0	0		*p *≤ 1
	P/S	7	22	8	25		*p *= 0.77
6	Q/Q	9	28	3	9	sign.*	not sign.
	K/K	15	47	22	69	*p *≤ 0.05	*p *≤ 0.2
	Q/K	8	25	7	22	*p *= 0.038	*p *= 0.11
7	L/L	8	25	3	9	sign.*	not sign.
	S/S	8	25	14	44	*p *≤ 0.05	*p *≤ 0.2
	L/S	16	50	15	47	*p *= 0.048	*p *= 0.14
Total		32		32			

^1#) ^Based on homozygous numbers

^2#) ^Based on homozygous and heterozygous numbers

**Table 4:** Primers used for genotyping of SNPs

**Fragment**	**Size**	**Primer**	**Nucleotide sequence (5'-3')**
Exon 7-8	511 bp	Exon7-8.F	AGGGGCAGATGGTATGTCAG
		Exon7-8.R	CAACCTCCCTCATTTCCAGA
Exon 9	165 bp	Exon9.F	CCCCTAGATTATGAAGAGGTGGA
		Exon9.R	TCTTTGAACCTGGACGGAAC

All primers were designed in this study from the sequence: GenBank: DQ823081
